# Supplementary material for: The pre-anesthetic period is the best time to evaluate the knee flexion angle for predicting the flexion angle after total knee arthroplasty: A prospective cohort study
Source: PLoS One. 2023 Feb 3;18(2):e0281237. doi: 10.1371/journal.pone.0281237 (PMC9897552; doi:10.1371/journal.pone.0281237)
Supplement: Attachment — Submitted filename: Response to Reviewers.docx [file pone.0281237.s001.docx]

**Response to reviewers**

May 2022, **Plos One**, Rapeepat Narkbunnam, Siriraj Hospital, Thailand

Dear Editor and reviewers,

We are very excited to have been given the opportunity to revise our manuscript "**The pre-anesthetic period is the best time to evaluate the knee flexion angle for predicting the flexion angle after total knee arthroplasty: A prospective cohort study**." We are pleased to submit the revised version for reconsideration for publication in **Plos One.**

We highly appreciate the detailed and valuable comments from the reviewers. The suggestions are helpful for us, and we shall incorporate most of them in the revised paper. As below, on behalf of my co-authors, I would like to clarify all the points raised by the reviewers, and we hope the reviewers and the Editor will be satisfied with our responses to the comments and the revisions of the original manuscript.

According to ethical and legal restrictions on sharing a sensitive dataset, we would like to change the statement of availability of data and material to “Data are available from the Siriraj Institutional Data Access / Ethics Committee (contact via orthoresearch.si@gmail.com) for researchers who meet the criteria for access to confidential data.”

Sincerely yours,

Rapeepat Narkbunnam, M.D.

Associated Professor in Orthopaedics Adult Reconstructive Surgery,
Department of Orthopaedics Surgery, Siriraj Hospital, Mahidol University, Thailand

Tel: +66(8)81-935-1669

E-mail address: mai_parma@hotmail.com

Reviewer #1: This prospective study compares the pre-anesthetic, intraoperative and posts anaesthetic KFA of patients undergoing TKR with the 6 month KFA concluding that the pre-anesthetic range more closely predicts the 6 month range.

 These results are not unexpected and have been reported before, with many showing that the final ROM is related to pre operative ROM. This study does add to the literature by delineating the 3 perioperative assessments with the final KFA and so does offer some useful information, however the ROM under anaesthetic should be higher than pre anaesthesia when pain often limits ROM.

**Response to Reviewer #1:**

We are delighted and thankful to hear that you think our work contributes scientific knowledge. Thank you for taking the time and energy to help us improve the paper. Below are our responses to your comments.

**There are some points that need to be clarified:**

 1. Why were the knees that required MUA excluded from the analysis? I would have expected them to be included.

RESPONSE: Thank you for your valuable comment. We apologize that our previous manuscript might not elaborate well enough on this issue and may cause ambiguity. We have thus provided a revision incorporating the rationale for our decision and the evidence supporting it.

We excluded TKA that required MUA because we intended to answer the research question only in primary TKA with no related surgical issues and no abnormal biologic responses. The cases requiring MUA or stiff TKA may have different recovery courses after TKA and are usually related to surgical or abnormal biological factors. A recent systematic review in 2021 from Zaffagnini et al. reported that various surgical errors and/or abnormal biologic responses are related to stiffness after TKA requiring MUA, e.g. internal rotation of the tibial component, malalignment of the femoral component, prosthetic overhang, excessive tension within the joint capsule, an abnormal biological factor related to individual phenotypic expression, and immune reactions to the metal. Also, patients who develop arthrofibrosis and psychological distress are significantly related to having stiff TKA. These factors could diminish or alter the course of gaining a KFA. Therefore, excluding TKA that required MUA would ensure that we can generalize the study findings to patients with regular biological responses without significant surgical error related to TKA.

We have included the rationale and the supporting evidence on this issue to improve the quality of the revised manuscript.

**Five lines number 142-146 have been added** to the revised manuscript in the material and methods session. **(Line 142-146, "We excluded TKA that required MUA because we intended to focus our study question in primary TKA with no stiffness related to the surgery and no abnormal biologic responses that may turn into arthrofibrosis. Such excluded cases may have different recovery courses after TKA that are usually related to surgical issues and/or abnormal biological responses. [15]”)**

Moreover, **two lines number 102-103 have also been added** to elaborate on this issue in the limitation section. **(Line 102-103, "Our results might not be generalizable to patients with secondary osteoarthritis, surgical issues related to stiffness, and abnormal biological responses causing arthrofibrosis."**)
And, we added **four lines number 336-339** (**Line 336-339, "First, we only included patients with primary knee osteoarthritis and excluded patients who required MUA after TKA. Therefore, our results might not be generalizable to patients with secondary osteoarthritis, surgical issues related to stiffness, and abnormal biological responses causing arthrofibrosis.”)**

2. The authors suggest that 6 months evaluation should show the endpoint improvement in KFA, but the cited reference was not indicating that as it was a study of only 25 patients with follow up limited to 6 months. most experienced surgeons would suggest that KFA can improve well past 1 year, even out to 2 years. Studies looking at PROMS show improvement past 1 year which is likely to be related to change in KFA.

RESPONSE: Thank you for reminding us of this critical point. We agreed with these valuable comments. We apologize that our previous manuscript might not provide an apparent rationale for choosing the follow-up time of 6 months in our study. Nevertheless, we did a revision by bringing out the point that there were existing evidence revealing that the improvement of TKA in terms of the range of motion can typically be seen since 3 to 6 months after TKA. **Three lines number 126-128 have been added, (Line 126-128, “Previous evidence have reported that the steady phase of knee ROM recovery after TKA could be reached at six-months post-operation [11-14].”)**  However, we also added further supportive evidence as per your recommendation to emphasize that fractional improvement can slowly occur and can be found beyond six months after TKA. **Four lines from 285-288 have been added, (Line 286-289, “Although some evidence suggested the plateau phase of recovery in knee motion can improve well past one year and even last up to 2 years [26-27], our study focuses on up to 6 months of follow-up. This was because the recovery after 6 months usually involves only a slight improvement compared to the magnitude of recovery during the first six months of follow-up. [12]”)** Finally, we also deleted the term used in the previous manuscript from "Plateau phase of recovery" to "6 months KFA follow-up" to avoid the confusion to the readers and conflicts with the evidence on the plateau phase of recovery after TKA. **(Edited term, line 331, from "which was reported the plateau phase of recovery" to "6-month KFA follow-up")**

3. The authors need to highlight that high KFA would be expected while the patient was anaesthetized, and so both the intraoperative and immediate postoperative measurements are really just an indication of creating an adequate flexion gap at the time of surgery. If the KFA is diminished at this stage then the flexion gap is too tight and will never recover.

RESPONSE: We want to thank the reviewer for these thoughtful comments, which help improve this manuscript's quality. **Four lines have been added** to the discussion part to highlight this vital message to the readers. **(Line 292-296, Our findings highlighted that high KFA would be expected while the patients were anesthetized. This means both the intraoperative and immediate postoperative measurements just represent an adequate flexion gap at the time of surgery. If the KFA is diminished at this stage, then the flexion gap is too tight and will not likely recover.**) We also add this piece of message to the key message. **(Line 91-94, "High KFA would be expected while the patients were anesthetized. This means both the intraoperative and immediate postoperative measurements just represent an adequate flexion gap at the time of surgery. If the KFA is diminished at this stage, then the flexion gap is too tight and will not likely recover.”)**

4. The authors indicate the error with measuring KFA but these are from other studies and the error for this study should be done separately

RESPONSE: Thank you for reminding us of this important issue. We agree that it would be helpful to clarify that the measurement error for this study, which may not be the same as the previous study. Unfortunately, we did not calculate the inter- and intra-rater reliability in this study. This was because the measurement method had been proven and well-defined according to Handcock GE, et al. However, we was also concerned about this issue, so we have provided more details in our revised manuscript. We emphasize how we tried to minimize the measurement error in the methods section. **(Line 185-188, "In the present study, measuring KFA at each time point was performed and recorded in the same manner. The recorded KFA was an average of the angle we retrieved after measuring consecutively twice by the measurer, who had been trained and tested for reliability before collecting the data.")**

We also described this issue as the reviewer's suggestion in the limitation part. **(Line 342-345, "Lastly, we did not analyze inter and intra-rater reliability. Nonetheless, we did attempt to minimize the measurement error of the KFA, and we believe that the measurement technique we applied in this study had been proven in the literature with detailed steps to follow.”)**
